# Supplementary material for: Label-free detection of conformational changes in switchable DNA nanostructures with microwave microfluidics
Source: Nat Commun. 2019 Mar 12;10:1174. doi: 10.1038/s41467-019-09017-z (PMC6414672; doi:10.1038/s41467-019-09017-z)
Supplement: Supplementary file 1 — Supplementary Information [file 41467_2019_9017_MOESM1_ESM.pdf]

Supplementary Information for “Label-Free Detection of Conformational Changes in Switchable DNA Nanostructures with Microwave Microfluidics”

Stelson *et al*

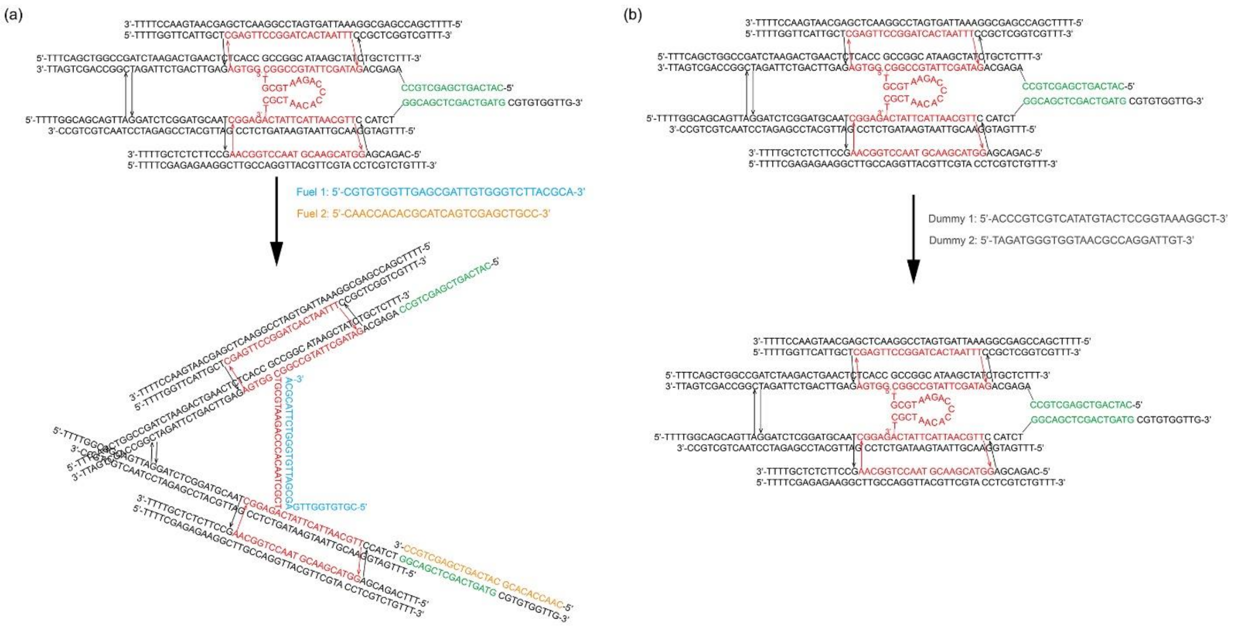

Supplementary Figure 1: Sequence design of closed tweezer, open tweezer and dummy strands. (a) Switch from a closed to an open tweezer upon addition of Fuel 1 (blue) and Fuel 2 (orange) strands. The closed tweezer was locked at the end by two complementary extensions (green) to facilitate close state. (b) Control tweezer with addition of non-complementary Dummy strands (gray); no conformational change is expected.

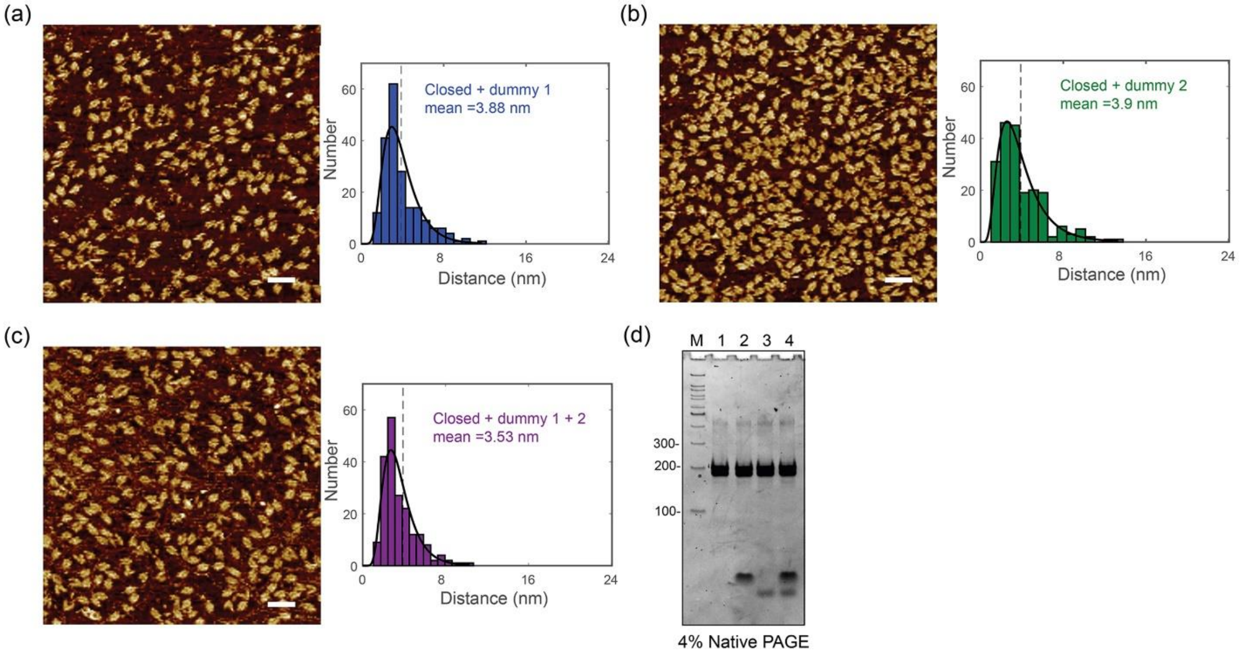

Supplementary Figure 2: AFM images and distance histograms and gel characterization of closed tweezers with dummy strands. (a) AFM image and distance histograms (dark blue) of the closed tweezer upon addition of dummy 1 strand. (b) AFM image and distance histograms (dark green) of the closed tweezer upon addition of dummy 2 strand. (c) AFM image and distance histograms (purple) of the closed tweezer upon addition of both dummy 1 and dummy 2 strands. Scale bars: 50 nm. (d) 4 % native PAGE gel characterization: lane M, dsDNA ladder (bp); lane 1, closed tweezer with locking strands; lane 2, closed tweezer with dummy 1 strand; lane 2, closed tweezer with dummy 2 strand; lane 3, closed tweezer with both dummy 1 and dummy 2 strands.

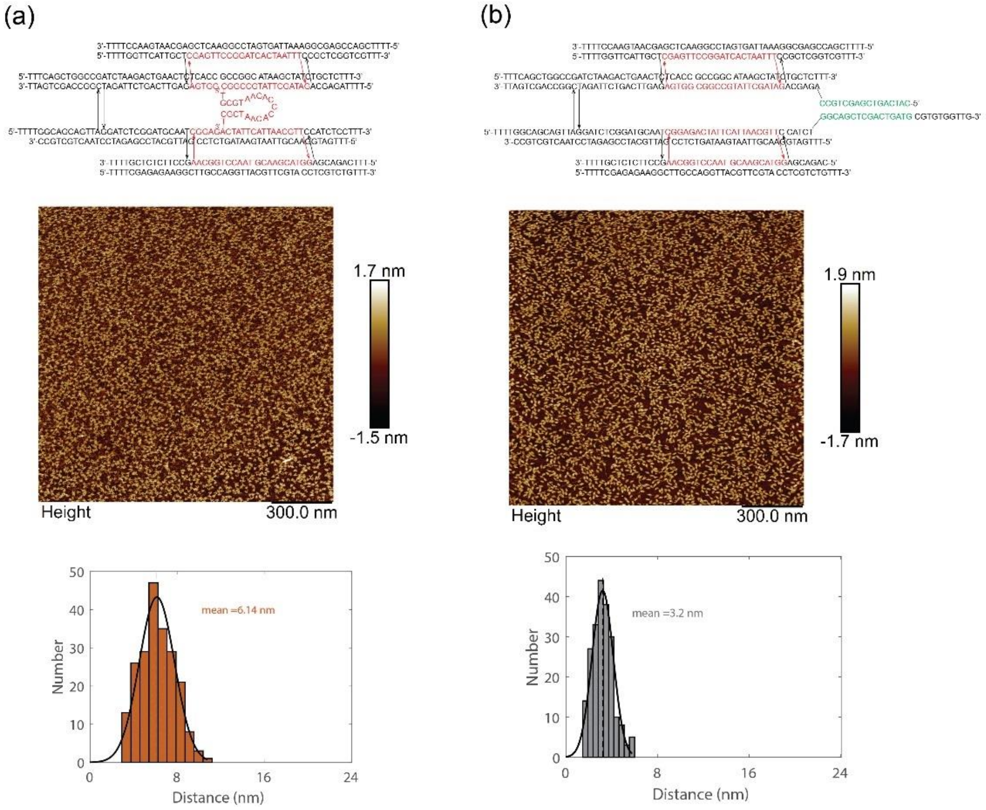

Supplementary Figure 3: AFM images and distance histograms and gel characterization of closed tweezers with dummy strands. (a) Sequence design, AFM images and distance histograms (orange) of the closed tweezer without the complementary extension locking mechanism. (b) Sequence design, AFM images and distance histograms (gray) of closed tweezer without the hairpin locking mechanism.

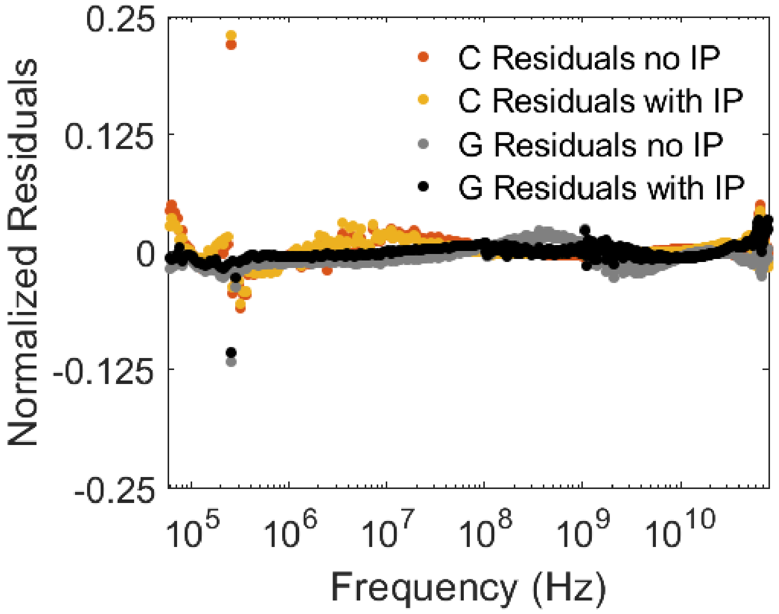

Supplementary Figure 4: Residuals normalized to the fit magnitude for C_{tot} and G_{tot} fits (for example, $\frac{(C_{\mathrm{tot}}-fit(C_{\mathrm{tot}}))}{fit(C_{\mathrm{tot}})}$) that include a Debye relaxation for ion-pairing (yellow and black) and do not include a Debye relaxation while allowing a Cole-Cole distribution for the water relaxation (orange and gray).

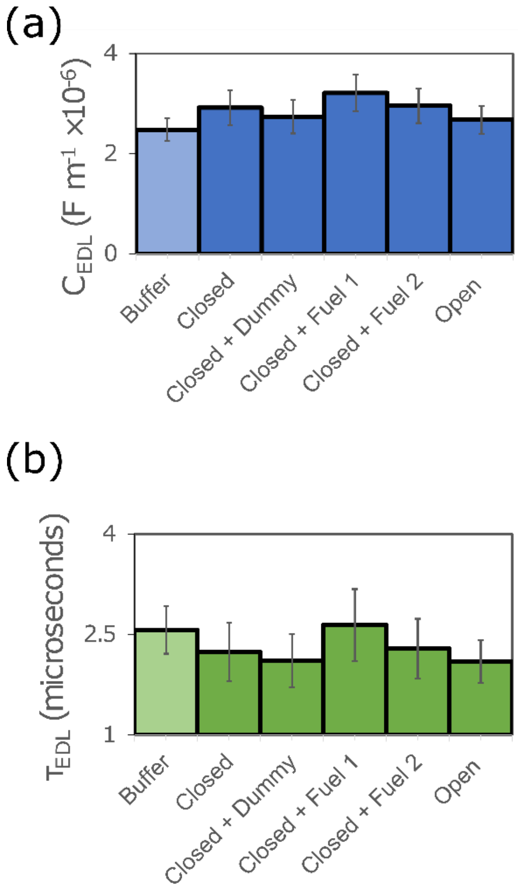

Supplementary Figure 5: (a) Dipolar contribution of EDL C_{EDL}. (b) Relaxation time of the EDL τ_{EDL}.

Error bars represent 95 % confidence intervals for N = 605 frequency points.

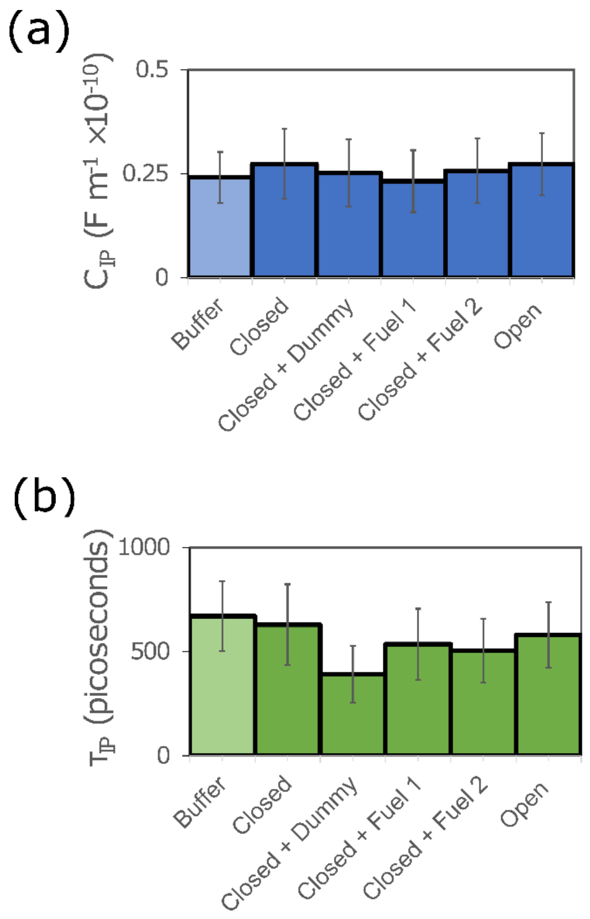

Supplementary Figure 6: (a) Dipolar contribution of ion pairs C_{IP}. (b) Relaxation time of ion pairs τ_{IP}. Error bars represent 95 % confidence intervals for N = 605 frequency points.

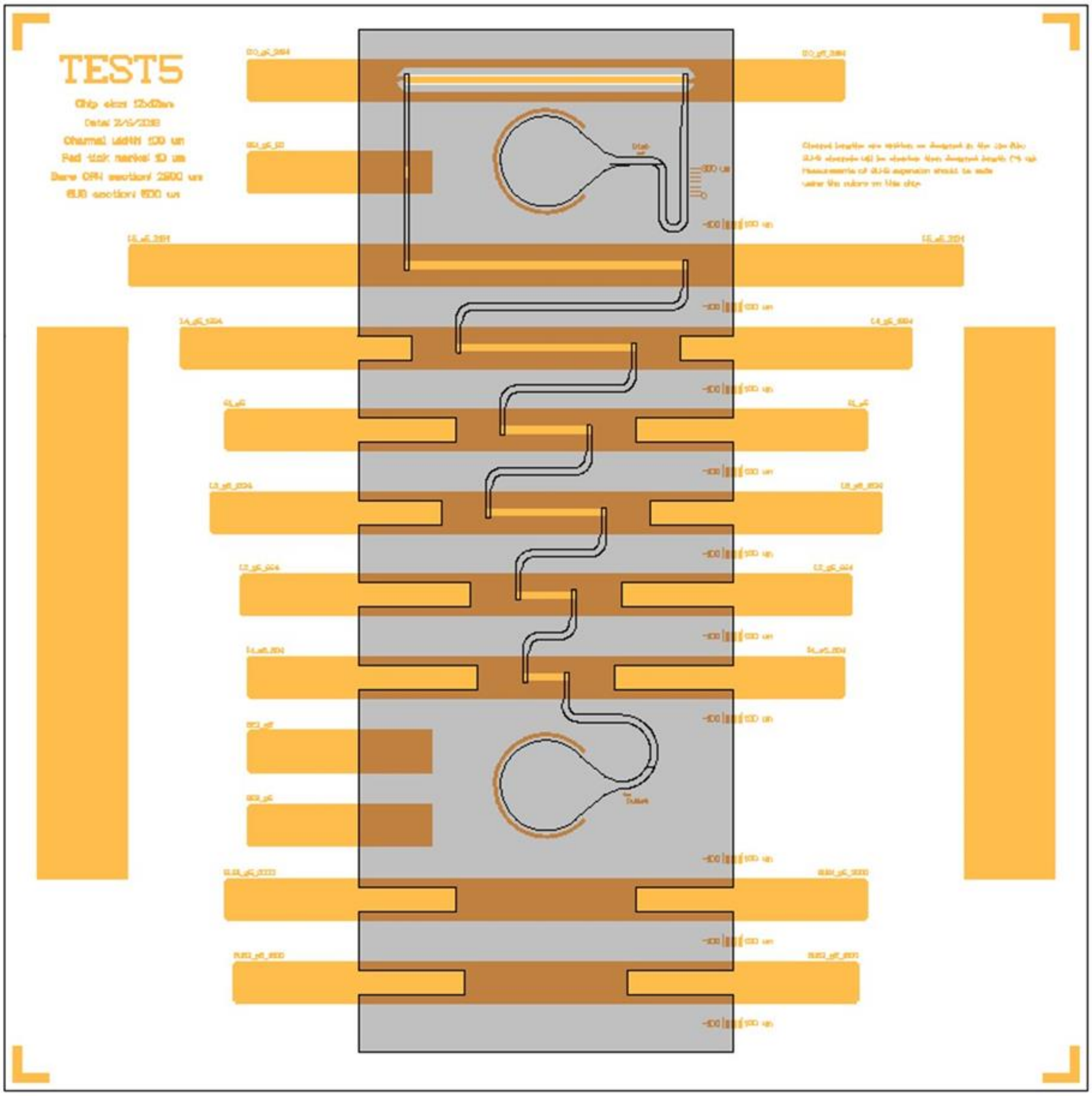

Supplementary Figure 7: Schematic of chip layout. We deposited gold (yellow regions), followed by SU-8 layers (gray regions). Channels in the PDMS passing over SU-8 are outlined in black.

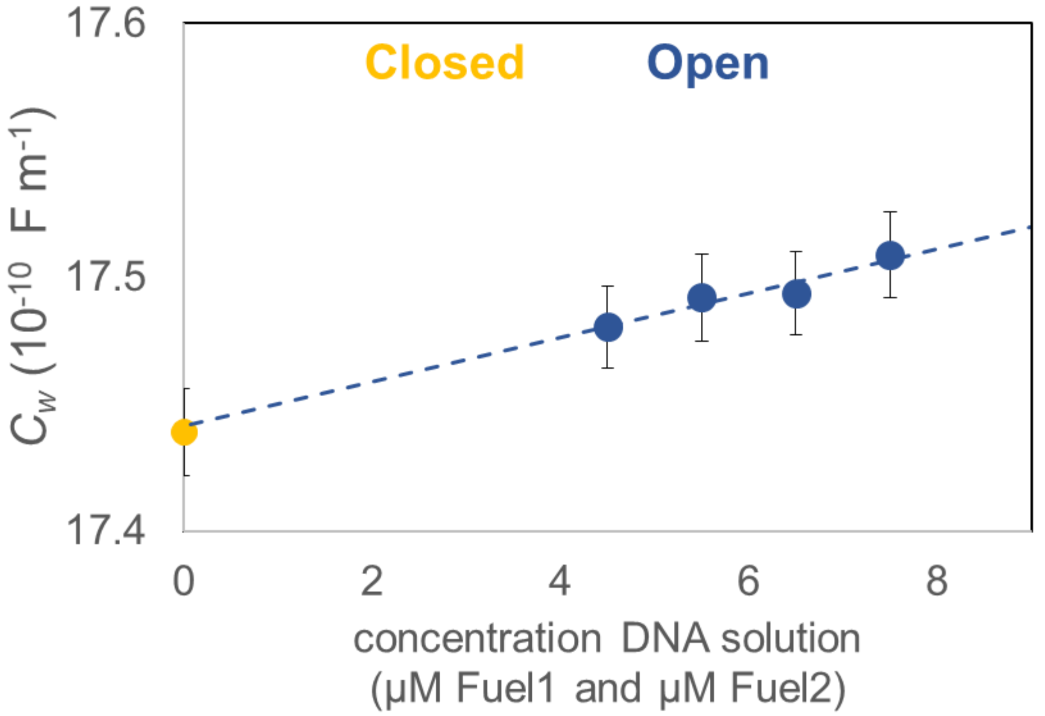

Supplementary Figure 8: Dipolar contribution of water C_{w} for closed (yellow) and (blue) open tweezers with varied concentration of excess fuel strands (Fuel 1 and Fuel 2). For reference, 5 µM concentration corresponds to 10x Fuel 1 and 10x Fuel 2 in solution. Note that these measurements are taken on a different device than the data in the main manuscript, and absolute magnitudes are not directly comparable. Error bars correspond to 95 % confidence intervals for N = 605 frequency points.

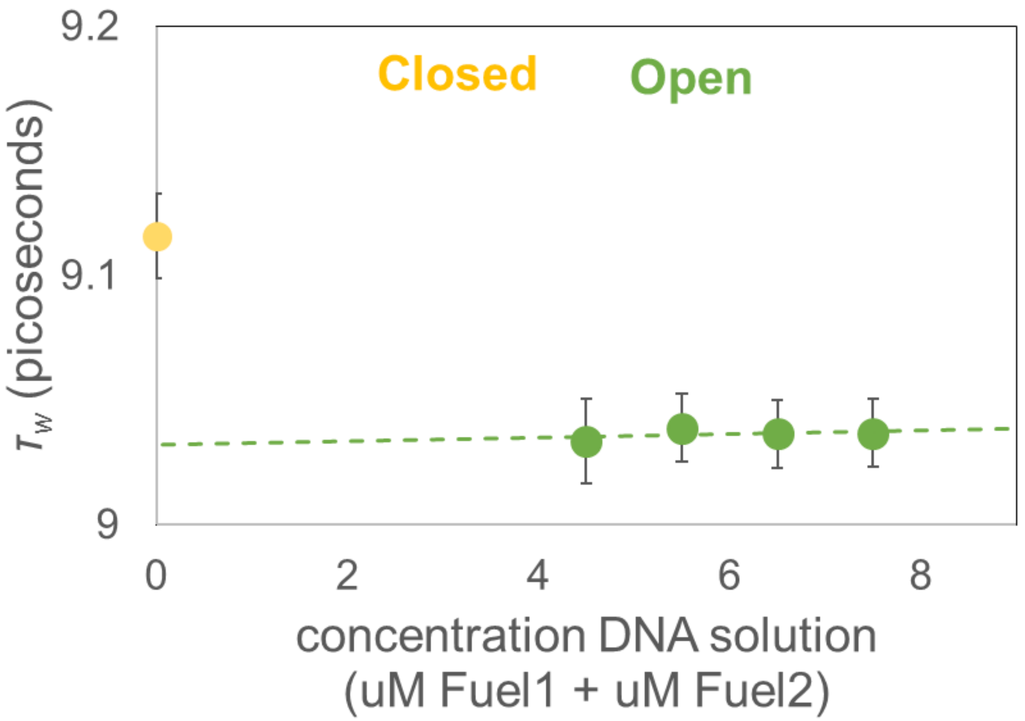

Supplementary Figure 9: Time constant of the cooperative water relaxation τ_w for closed (yellow) and (green) open tweezers with varied concentration of excess fuel strands (FUEL 1 and FUEL 2). For reference, 5 µM concentration corresponds to 10x FUEL 1 and 10x FUEL 2 in solution. Note that these measurements are taken on a different device than the data in the main manuscript, and absolute magnitudes are not directly comparable. Error bars correspond to 95 % confidence intervals for N = 605 frequency points.

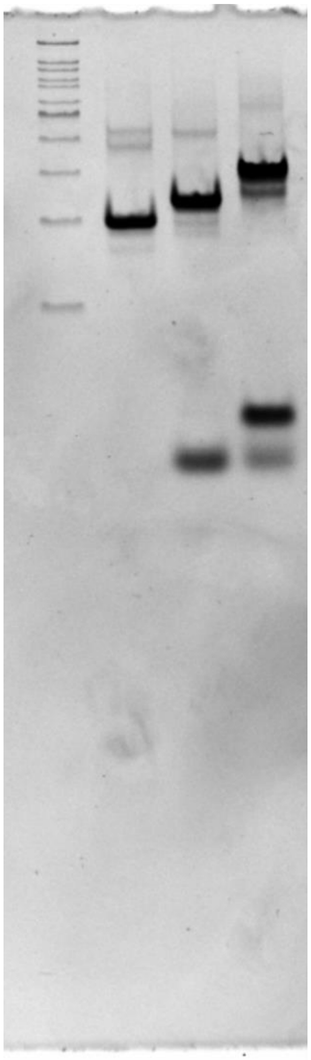

Supplementary Figure 10: Original 4% native PAGE gel for Figure 1 (f).

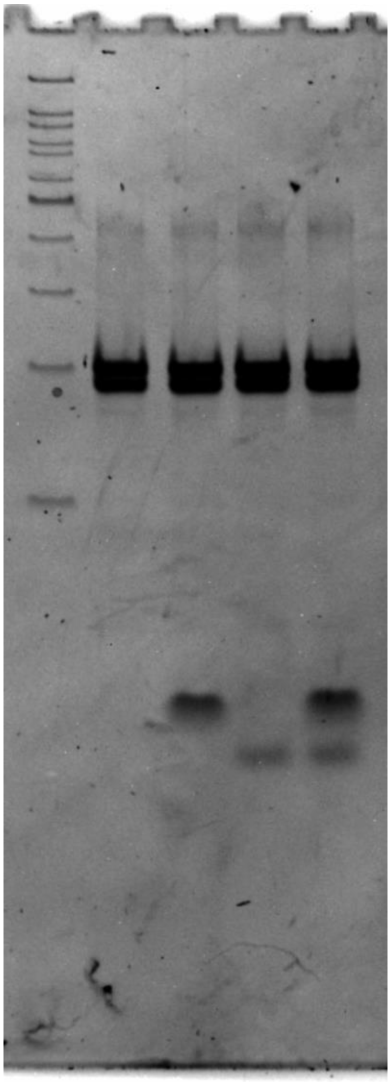

Supplementary Figure 11: Original 4% native PAGE gel for Supplementary Figure 2 (d).

Supplementary Table 1: Fit parameters for ionic conductivity and water relaxation. All fit parameters are presented with 95 % confidence intervals for N = 605 frequency points. All p-values are for Z-tests with unequal variance.

**Sample**	$\boldsymbol{G_\sigma}$ (S m^{-1})	*P*-value for $\boldsymbol{G_\sigma}$ (test against closed)	$\boldsymbol{C_\mathrm{w}}$ (10^{-10} F m^{-1})	*P*-value for $\boldsymbol{C_\mathrm{w}}$ (test against closed)	$\boldsymbol{C_\infty}$ (10^{-10} F m^{-1})	*P*-value for $\boldsymbol{C_\mathrm{w}-C_\infty}$ (test against closed)	$\boldsymbol{\tau_\mathrm{w}}$ (ps)	*P*-value for $\boldsymbol{\tau_w}$ (test against closed)
DI water	0	$p < 10^{-5}$	17.43 ± 0.007	$p < 10^{-5}$	2.30 ± 0.006	$p < 10^{-5}$	8.33 ± 0.005	$p < 10^{-5}$
EDTA Buffer	0.64 ± 0.002	$p < 10^{-5}$	17.29 ± 0.007	$p < 10^{-5}$	2.20 ± 0.007	$p < 10^{-5}$	8.37 ± 0.005	$p < 10^{-5}$
Closed Tweezers	0.85 ± 0.004		17.24 ± 0.008		2.20 ± 0.007		8.44 ± 0.005	$p = 0.40$
Closed + Dummy	0.83 ± 0.004	$p < 10^{-5}$	17.24 ± 0.008	$p = 0.38$	2.20 ± 0.007	$p = 0.58$	8.43 ± 0.005	$p < 10^{-3}$
Closed + Fuel 1	0.83 ± 0.004	$p < 10^{-5}$	17.22 ± 0.008	$p < 10^{-5}$	2.20 ± 0.007	$p = 0.034$	8.43 ± 0.005	$p < 10^{-5}$
Closed + Fuel 2	0.82 ± 0.003	$p < 10^{-5}$	17.24 ± 0.008	$p = 0.85$	2.21 ± 0.007	$p = 0.41$	8.44 ± 0.005	$p = 0.08$
Open Tweezers	0.79 ± 0.003	$p < 10^{-5}$	17.26 ± 0.008	$p = 0.018$	2.20 ± 0.007	$p = 0.028$	8.42 ± 0.005	$p < 10^{-3}$

Supplementary Table 2: Fit parameters for the ion-pairing relaxation, the electrical double layer and the constant phase element. All fit parameters are presented with 95 % confidence intervals for N = 605 frequency points. All p-values are for Z-tests with unequal variance.

**Sample**	C_{IP} (10^{-10} F m^{-1})	τ_{IP} (ps)	C_{EDL} (10^{-10} F m^{-1})	τ_{EDL} (ps)	$1-\alpha_{\mathrm{EDL}}$	Q (S m^{-1} Hz^{-1})
DI water	0	0	0	0	0	0
EDTA Buffer	0.24 ± 0.06	670 ± 194	$(2.4 \pm 0.2) \times 10^4$	$(2.6 \pm 0.4) \times 10^6$	0.76 ± 0.03	$(1.0 \pm 0.0) \times 10^4$
Closed Tweezers	0.27 ± 0.08	630 ± 167	$(2.9 \pm 0.4) \times 10^4$	$(2.2 \pm 0.4) \times 10^6$	0.69 ± 0.04	$(1.0 \pm 0.0) \times 10^4$
Closed + Dummy	0.25 ± 0.08	392 ± 137	$(2.7 \pm 0.3) \times 10^4$	$(2.1 \pm 0.4) \times 10^6$	0.71 ± 0.04	$(1.0 \pm 0.0) \times 10^4$
Closed + Fuel 1	0.26 ± 0.08	505 ± 152	$(3.0 \pm 0.3) \times 10^4$	$(2.3 \pm 0.4) \times 10^6$	0.69 ± 0.03	$(1.0 \pm 0.0) \times 10^4$
Closed + Fuel 2	0.23 ± 0.07	536 ± 170	$(3.2 \pm 0.4) \times 10^4$	$(2.6 \pm 0.6) \times 10^6$	0.67 ± 0.03	$(1.0 \pm 0.0) \times 10^4$
Open Tweezers	0.27 ± 0.07	580 ± 156	$(2.7 \pm 0.3) \times 10^4$	$(2.1 \pm 0.3) \times 10^6$	0.74 ± 0.03	$(1.1 \pm 0.0) \times 10^4$

Supplementary Methods

DNA strands: Single-stranded oligonucleotides were purchased from IDT DNA (Integrated DNA Technologies, Inc.).

Buffers: Tris base, acetic acid, EDTA, and magnesium acetate were purchased from Sigma Aldrich.

Design, assembly, and characterization of DNA nanotweezers

DNA nanostructure design: The detailed sequence designs of the original DNA nanotweezers and photocaged DNA nanotweezers are shown in Supplementary Figure 1. Tiamat (downloaded from http://yanlab.asu.edu/Resources.html) was used for structure and sequence design. All sequences are written in 5'-3' order.

Sequences of closed DNA nanotweezers:

T1: TTTTCGACCGAGCGGAAATTAGTGATCCGGAACTCGAGCAATGAACCTTTT

T2: TTTCAGCTGGCCGATCTAAGACTGAACTCTCACCGCCGGCATAAGCTATCTGCTCTTT

T3: TTTGATGGAACGTTAATGAATAGTCTCCGATTGCATCCGAGATCCTAACTGCTGCC

T4: TTTTCGAGAGAAGGCTTGCCAGGTTACGTTCGTACCTCGTCTGTTT

T5: TTTTGGCAGCAGTTACGGCCAGCTGATT

T6: TTTTGGTTCATTGCTGAGTTCAGTCTTAGATGGATCTCGGATGCAATGCCTTCTCTCGTTTT

T7: GGTGACGAGTTCCGGATCACTAATTTGATAGCTTATGCCGGCTGCGTAAGACCCACAATCGCTACTATTCATTAACGTTGGTACGAACGTAACCTGGCAACGGAG

T8-lock: CATCAGTCGAGCTGCCAGAGCACCGCTCGGTCGTTT

T9-lock-toehold: CAGACGACCATCT GGCAGCTCGACTGATG CGTGTGGTTG

Sequences of dummy strands:

Dummy 1: ACCCGTCGTCATATGTACTCCGGTAAAGGCT

Dummy 2: TAGATGGGTGGTAACGCCAGGATTGT

Sequences of open DNA nanotweezers (with 2 fuel strands):

T1: TTTTCGACCGAGCGGAAATTAGTGATCCGGAACTCGAGCAATGAACCTTTT

T2: TTTCAGCTGGCCGATCTAAGACTGAACTCTCACCGCCGGCATAAGCTATCTGCTCTTT

T3: TTTGATGGAACGTTAATGAATAGTCTCCGATTGCATCCGAGATCCTAACTGCTGCC

T4: TTTTCGAGAGAAGGCTTGCCAGGTTACGTTCGTACCTCGTCTGTTT

T5: TTTTGGCAGCAGTTACGGCCAGCTGATT

T6: TTTTGGTTCATTGCTGAGTTCAGTCTTAGATGGATCTCGGATGCAATGCCTTCTCTCGTTTT

T7: GGTGACGAGTTCCGGATCACTAATTTGATAGCTTATGCCGGCTGCGTAAGACCCACAATCGCTACTATTCATTAACGTTGGTACGAACGTAACCTGGCAACGGAG

T8-lock: CATCAGTCGAGCTGCCAGAGCACCGCTCGGTCGTTT

T9-lock-toehold: CAGACGACCATCT GGCAGCTCGACTGATG CGTGTGGTTG

Fuel-1: CGTGTGGTTGAGCGATTGTGGGTCTTACGCA

Fuel-2: CAACCACACGCATCAGTCGAGCTGCC

Purification of DNA oligonucleotides: DNA oligonucleotides were purified in the lab using denaturing polyacrylamide gel electrophoresis (PAGE) [1, 2]. 8-10% denaturing PAGE gels (with 8.3M urea) can be prepared by mixing 20% and 0% polyacrylamide gel mix solutions at room temperature. Ammonium persulphate (APS) is used as gel polymerization initiator and N,N,N',N'-tetramethylethane-1,2-diamine (TEMED) is used as cross-linker. DNA oligonucleotides were loaded in wells and run for ~1.5 hours at 40 ℃ at a constant current of 45 mA per gel. The gel was placed on a TLC silica plate and exposed under 254 nm UV light. The bands corresponding to the correct length were cut out from the gel, chopped and transferred into Costar Spin X filtration column (Corning, cellulose acetate membrane with 0.22 µm size) and incubated for overnight in elution buffer (500 mM ammonium acetate, 10 mM magnesium acetate, 2 mM sodium ethylenediaminetetraacetic acid, pH 8.0). On the next day, DNA strands were extracted using ethanol precipitation and then dissolved in nanopure water. Concentrations of DNA strands can be measured by UV absorbance at 260 nm and calculated using extinction coefficient provided by IDT.

DNA nanotweezers assembly: The DNA strands that constitute each DNA structure were combined in an equimolar ratio in 1×TAE-Mg^{2+} buffer (40 mM Tris, 20 mM acetic acid, 2 mM EDTA and 12.5 mM magnesium acetate, pH 8.0) to reach a final concentration of 0.5 µM per strand. Fuel and dummy strands were added with 10-fold excess and all samples (actuated, unactuated, unactuated with dummy strands) stored 1 day to 2 days at 4 °C prior to measurement.

Supplementary References

1) M. H. Liu, J. L. Fu, C. Hejesen, Y. H. Yang, N. W. Woodbury, K. Gothelf, Y. Liu, H. Yan, Nat Commun 2013, 4.

2) J. L. Fu, Y. R. Yang, A. Johnson-Buck, M. H. Liu, Y. Liu, N. G. Walter, N. W. Woodbury, H. Yan, Nat Nanotechnol 2014, 9, 531-536.
